# A meta-analysis of randomized double-blind clinical trials in CMT1A to assess the change from baseline in CMTNS and ONLS scales after one year of treatment

**DOI:** 10.1186/s13023-015-0293-y

**Published:** 2015-06-13

**Authors:** Jonas Mandel, Viviane Bertrand, Philippe Lehert, Shahram Attarian, Laurent Magy, Joëlle Micallef, Ilya Chumakov, Catherine Scart-Grès, Mickael Guedj, Daniel Cohen

**Affiliations:** Pharnext SAS, Issy-les-Moulineaux, France; Faculty of Medicine, University of Melbourne, Melbourne, Australia; Faculty of Economics, UCL Mons, Louvain, Belgium; Centre de référence des maladies neuromusculaires et de la SLA, Pôle des neurosciences Cliniques, AP-HM et Aix Marseille Université, Marseille, France; CHU de Limoges Hôpital Dupuytren, Limoges, France; CIC-Centre de Pharmacologie Clinique et D’Evaluations Thérapeutiques, AP-HM et Aix Marseille Université, Marseille, France

**Keywords:** Charcot-Marie-Tooth, CMT1A, CMTNS, ONLS, PXT3003, Meta-analysis, Ascorbic acid, Clinical trials, Randomized, Double blind

## Abstract

CMT1A is the most common inherited peripheral neuropathy. There is currently no approved treatment. We performed a meta-analysis including four randomized, double-blind, Placebo-controlled clinical trials to assess the disease progression after one year under Placebo, Ascorbic Acid (AA) or PXT3003, a combination of three repurposed drugs. We observed a weak deterioration in patients under Placebo, well below the reported natural disease progression. Patients treated with AA were stable after one year but not significantly different from Placebo. Patients undergoing PXT3003 treatment showed an improvement in CMTNS and ONLS, statistically significant versus Placebo and potentially precursory of a meaningful change in the disease course.

## Letter to the editor

Charcot-Marie-Tooth disease Type 1A (CMT1A, OMIM: 118220, Orphanet: ORPHA101081) is a rare, inherited, peripheral neuropathy caused by duplication of the gene PMP22 [1, 2], whose over-expression induces dysmyelination, axonal loss and muscle wasting [3, 4]. Two treatments have been recently investigated in seven 1- or 2-year randomized, double blind, placebo-controlled clinical trials: Ascorbic Acid (AA) [5–12] and PXT3003, a combination of (RS)-baclofen, naltrexone hydrochloride and D-sorbitol [13, 14]. Now that all these trials have been completed, and as recommended at the 168^th^ ENMC international workshop [15], we report the results of a first meta-analysis assessing the disease progression after one year under Placebo, AA or PXT3003.

## Methods

We conducted a literature search through PubMed and ClinicalTrials.gov for randomized, placebo-controlled clinical trials lasting 12 months or more using ‘‘Charcot-Marie-Tooth type 1A disease’’ and its synonyms’ ‘‘hereditary motor and sensory neuropathy’’, ‘‘peroneal muscular atrophy’’ and ‘‘distal spinal muscular atrophy’’ as the search terms. MEDLINE search terms are given in Appendix. We also checked the bibliography of identified trials. The outcomes of interest were the change from baseline in CMTNS [16] and ONLS [17] after one year of treatment or Placebo, hence only trials measuring CMTNS or ONLS were selected. CMTNS and ONLS are considered as the main clinical scales for impairment and disability, respectively, in CMT1A disease [15]. Studies measuring at least one of these two outcomes were selected. In both measures, an increasing score is considered as deterioration.

The estimated mean changes from baseline and corresponding standard errors were extracted from the publications. When not available, standard errors were deduced from confidence intervals. Studies not providing sufficient information were excluded from the meta-analysis.

For each outcome, we performed fixed and DerSimonian-Laird random effects meta-analyses including treatment (Placebo, AA or PXT3003) as moderator factor. The Q-test and I^2^ index were used to determine the level of heterogeneity in the random effect model. Comparisons of AA and PXT3003 versus Placebo were performed with tests of contrast of the moderator factor.

## Results

Four studies met the inclusion criteria: three on AA [10–12] and one on PXT3003 [14]. For the PXT3003 trial, only the dose showing a significant effect was considered, *i.e.* the highest dose tested termed ‘PXT3003 HD’. For ONLS in the Pareyson study, values at 24 months were used as values at 12 months were not available. In total, 565 patients were included in these trials: 220 with Placebo, 326 with AA (1, 1.5, 3 or 4 g per day) and 19 with PXT3003 (HD). The Q and I^2^ indices for the random effect models did not reveal significant heterogeneity for CMTNS (Q-test *p* = 0.28; I^2^ = 10.9 %) nor for ONLS (Q-test *p* = 0.36; I^2^ = 11.2 %), justifying reporting the results of the fixed effect models only.

**Fig. 1:**
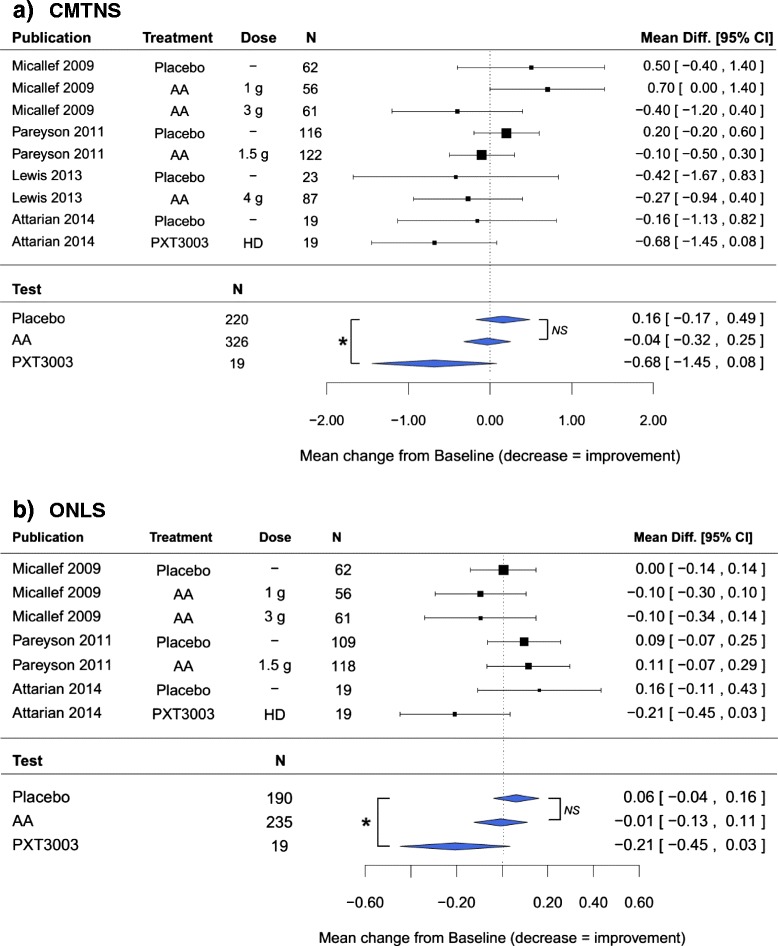
Results of the meta-analysis on the change from baseline after one year. Fixed-effect meta-analysis, with treatment as moderator variable. Difference in changes from baseline between Placebo, AA and PXT3003 were assessed through contrast tests. **a** Change from baseline in CMTNS under Placebo, AA and PXT3003; **b** Change from baseline in ONLS under Placebo, AA and PXT3003. *p < 0.05; NS = not-significant

Results obtained for CMTNS and ONLS scales were consistent (Fig. 1a and b). After one year, CMT1A patients showed a slight deterioration under Placebo of 0.16 point in CMTNS and 0.06 point in ONLS. The progression of patients under AA appeared stable (−0.04 point in CMTNS and −0.01 point in ONLS) and not significant when compared to Placebo (*p* = 0.390 for CMTNS and *p =* 0.387 for ONLS). Patients taking PXT3003 showed an amelioration in both measures (−0.68 point in CMTNS and −0.21 point in ONLS), significant when compared to Placebo (*p* = 0.048 for CMTNS and *p =* 0.044 for ONLS).

## Discussion

The present meta-analysis supports the conclusions made independently within each clinical trial as regards efficacy of treatments and Placebo [10, 12, 14]. First, the CMT1A patients of the Placebo groups from studies conducted from 2006 to 2014 deteriorate rather slowly compared to the estimated natural progression of 0.686 point/year in CMTNS reported by Shy and colleagues in 2008 [18]. These findings are consistent with the positive placebo effects observed in diabetic neuropathy [19] or patient-reported pain outcomes [20], although the factors accounting for such a difference remain unclear. Lewis *et al.* [12] considered that systematic differences between participants of the different studies may be partially responsible; for instance the mean age and CMTNS are slightly higher in the four clinical trials considered here than in the natural progression study by Shy *et al.* [18]. Pareyson and colleagues [11] also pointed out that the natural progression study was partly retrospective, and therefore might not be directly comparable with clinical trials. Consequently, we believe that the progression of CMTNS and ONLS under Placebo reported here is more valuable than natural progression estimates for the design of future clinical trials in CMT1A, and less prone to sampling bias that might occur in single independent studies.

Second, the progression of patients under different dosages of AA appears quite stable, and does not reach statistical significance versus Placebo. The difference between AA and Placebo is far below the order of magnitude expected for sample size calculation in the three AA clinical trials. As it happens, the *a posteriori* power to detect this difference as significant does not exceed 15 % (assuming an SD in CMTNS of 5, a correlation between baseline and final values of 0.8, and an ANCOVA analysis at a two-sided 5 % level). In this context, designing a confirmatory Phase 3 study for a treatment showing such stabilization in CMT1A would require a much larger sample size and longer study duration, making it clearly unrealizable. It confirms the idea that an effective treatment for this disease should bring an improvement, rather than the mere ability to slow or stabilize the disease progression [12, 14]. Even if this effect seems quite marginal, a standardized re-analysis of all AA patient-level data would be of great interest.

Lastly, this meta-analysis supports an improvement in both CMTNS and ONLS with PXT3003 treatment, statistically significant when compared to Placebo. This improvement could herald an early, meaningful change in the disease course.

Conducting a meta-analysis of clinical trials in CMT1A is challenging because of the small number of studies and of the heterogeneity of study protocols in terms of recruitment criteria, study duration, balance of groups, and statistical analysis. In addition, our study evaluates CMTNS in a context where a second version (CMTNSv2) has been proposed to reduce floor/ceiling effects and eventually to improve the scale’s sensitivity to change [21]. The current version of the CMTNSv2 has also been questioned recently through a Rasch analysis by Sadjadi *et al.* [22] and a ‘weighted’ alternative has been suggested. In parallel, Mannil *et al.* [23] proposed a CMTNSMod by adding three functional measures (9-hole peg test, foot dorsiflexion and walk test) while removing Ulnar SNAP, Pin Sensibility, Vibration and Strength of Arms. None of these modified versions has been evaluated yet in natural history or therapeutic trials. Despite these limitations, the present study provides a set of relevant observations, consistently obtained on both CMTNS and ONLS, to be used for the design of future clinical trials in CMT1A.

## Appendix

### PubMed MEDLINE Search Strategy

randomized controlled trial [Title/Abstract] OR controlled clinical trial [Title/Abstract] OR placebo-controlled clinical trial [Title/Abstract] OR clinical trial [Title/Abstract] OR randomized clinical trials [mh] OR clinical trials [mh] OR random allocation [mh] OR double-blind [mh] OR double blind[mh] OR ((singl* [tw] OR doubl* [tw] OR trebl* [tw] OR tripl* [tw]) AND (mask* [tw] OR blind* [tw])) OR (placebos [mh] OR placebo* [tw] OR random* [tw] OR follow-up study [mh] OR prospective study [mh])) NOT (animals [mh] NOT human [mh])

AND ((Charcot-Marie-Tooth [Title/Abstract] OR CMT [Title/Abstract] OR hmsn [Title/Abstract] OR hereditary motor and sensory neuropathy [Title/Abstract] OR peroneal muscular atrophy [Title/Abstract]) AND (1A [Title/Abstract] OR type 1A [Title/Abstract]))

## Abbreviations

CMT1A: Charcot-Marie-Tooth disease type 1A
CMTNS: Charcot-Marie-Tooth neuropathy score
ONLS: Overall neuropathy limitation scale
AA: Ascorbic acid
SD: Standard deviation
